# Future space experiment platforms for astrobiology and astrochemistry research

**DOI:** 10.1038/s41526-023-00292-1

**Published:** 2023-06-12

**Authors:** Andreas Elsaesser, David J. Burr, Paul Mabey, Riccardo Giovanni Urso, Daniela Billi, Charles Cockell, Hervé Cottin, Adrienne Kish, Natalie Leys, Jack J. W. A. van Loon, Eva Mateo-Marti, Christine Moissl-Eichinger, Silvano Onofri, Richard C. Quinn, Elke Rabbow, Petra Rettberg, Rosa de la Torre Noetzel, Klaus Slenzka, Antonio J. Ricco, Jean-Pierre de Vera, Frances Westall

**Affiliations:** 1 Freie Universitaet Berlin, Department of Physics, Arnimallee 14, 14195 Berlin, Germany; 2 INAF-Osservatorio Astrofisico di Catania, Via Santa Sofia 78, 95123 Catania, Italy; 3 Department of Biology, University of Rome Tor Vergata, Via della Ricerca Scientifica, 00133 Rome, Italy; 4 UK Centre for Astrobiology, School of Physics and Astronomy, University of Edinburgh, Edinburgh, UK; 5 Univ Paris Est Creteil and Université Paris Cité, CNRS, LISA, F-94010 Créteil, France; 6 Muséum National d’Histoire Naturelle (MNHN), Molécules de Communication et Adaptation des Microorganismes (MCAM), CNRS, 57 rue Cuvier, 75005 Paris, France; 7 Interdisciplinary Biosciences Group, Belgian Nuclear Research Centre, SCK CEN, 2400 Mol, Belgium; 8 Dutch Experiment Support Center (DESC), Department of Oral and Maxillofacial Surgery/Oral Pathology, Amsterdam Bone Center (ABC), Amsterdam UMC Location VU University Medical Center (VUmc) & Academic Centre for Dentistry Amsterdam (ACTA), Gustav Mahlerlaan 3004, 1081 LA Amsterdam, The Netherlands; 9 Centro de Astrobiología (CAB), CSIC-INTA, Carretera de Ajalvir km 4, 28850 Torrejón de Ardoz, Madrid, Spain; 10 Institute of Hygiene, Microbiology and Environmental Medicine, Medical University of Graz, Neue Stiftingtalstraße 6, 8010 Graz, Austria; 11 Department of Ecological and Biological Sciences (DEB), University of Tuscia, Largo dell’Università snc, 01100 Viterbo, Italy; 12 NASA Ames Research Center, Moffett Field, CA USA; 13 German Aerospace Center (DLR), Institute of Aerospace Medicine, Radiation Biology Department, Linder Höhe, 51147 Cologne, Germany; 14 Instituto Nacional de Técnica Aeroespacial (INTA), Departamento de Observación de la Tierra, 28850 Torrejón de Ardoz, Madrid, Spain; 15 KS-3D–3D-Printing and Laser Services, In der Heide 16, 27243 Gross Ippener, Germany; 16 German Aerospace Center (DLR), Space Operations and Astronaut Training, Microgravity User Support Center (MUSC), Linder Höhe, 51147 Cologne, Germany; 17 Centre National de la Recherche Scientifique (CNRS), Centre de Biophysique Moléculaire, Orléans, France

**Keywords:** Environmental sciences, Microbiology, Techniques and instrumentation, Evolution, Biogeochemistry

## Abstract

Space experiments are a technically challenging but a scientifically important part of astrobiology and astrochemistry research. The International Space Station (ISS) is an excellent example of a highly successful and long-lasting research platform for experiments in space, that has provided a wealth of scientific data over the last two decades. However, future space platforms present new opportunities to conduct experiments with the potential to address key topics in astrobiology and astrochemistry. In this perspective, the European Space Agency (ESA) Topical Team Astrobiology and Astrochemistry (with feedback from the wider scientific community) identifies a number of key topics and summarizes the 2021 “ESA SciSpacE Science Community White Paper” for astrobiology and astrochemistry. We highlight recommendations for the development and implementation of future experiments, discuss types of in situ measurements, experimental parameters, exposure scenarios and orbits, and identify knowledge gaps and how to advance scientific utilization of future space-exposure platforms that are either currently under development or in an advanced planning stage. In addition to the ISS, these platforms include CubeSats and SmallSats, as well as larger platforms such as the Lunar Orbital Gateway. We also provide an outlook for in situ experiments on the Moon and Mars, and welcome new possibilities to support the search for exoplanets and potential biosignatures within and beyond our solar system.

## Introduction

More than two decades of experiments on the ISS have had, and continue to have, a strong impact on research, science, and society as a whole^1–3^. The growing number of astrobiology and astrochemistry experiments onboard the ISS provides new insights and knowledge, which, via new products, techniques, and technology, have a long-lasting effect on our daily lives and culture^4^. Astrobiology and astrochemistry address some of the most exciting questions to be asked by humankind, including the origins of life on Earth, life elsewhere in the universe or the exploration and colonization of other planets. A major topic in astrobiology and astrochemistry is radiation and the influence of the space environment or planetary conditions on biological systems and molecules. While laboratory facilities can simulate some individual parameters, it is not currently possible to faithfully replicate the space environment on the ground. In this respect, the ISS provides an excellent platform to perform irradiation experiments beyond the protective atmosphere of the Earth. Beyond the ISS, the design and implementation of new platforms (such as small satellite platforms, CubeSats^5–9^ or the Lunar Orbital Gateway^10^) offer new possibilities for experiments in space. The latter will rely heavily on machine learning and other advances in artificial intelligence, in particular for navigation^11,12^ and on-the-fly repair of hardware^13^, a trend that will surely continue in the future.

**Fig. 1:**
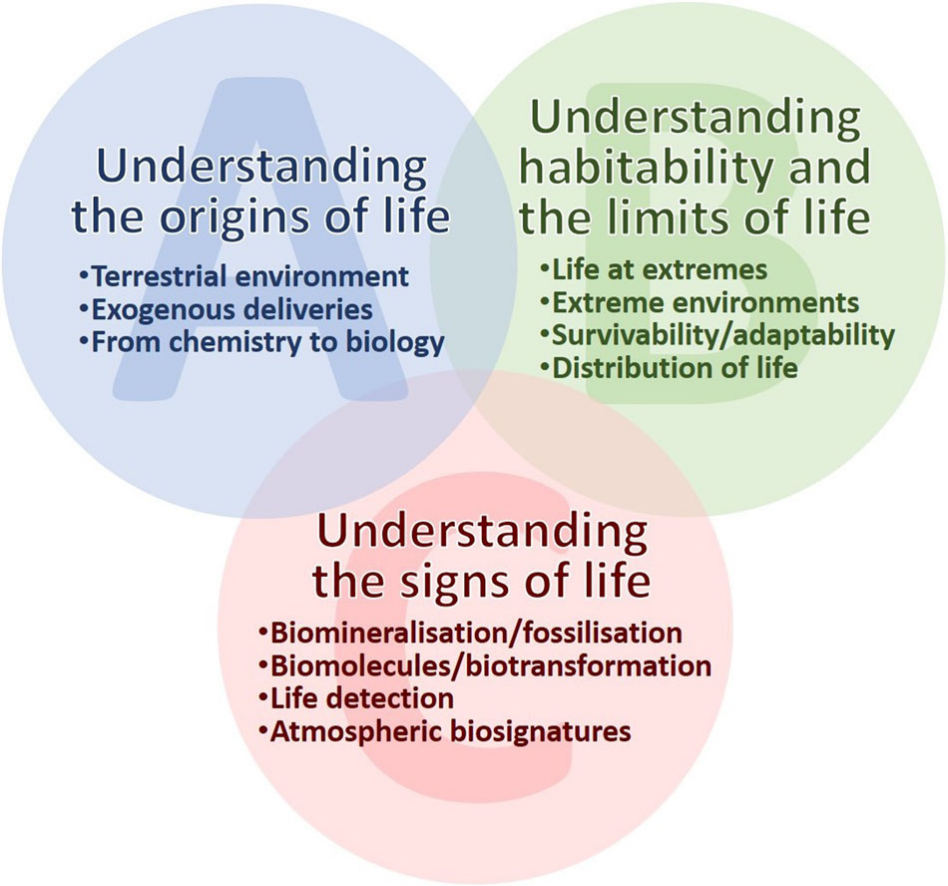
Key astrobiology and astrochemistry topics. As identified in the 2021 ESA SciSpacE Science Community White Paper (esamultimedia.esa.int/docs/HRE/10_Biology_Astrobiology.pdf), key astrobiology and astrochemistry topics are **A** understanding the origins of life, **B** understanding the habitability of life and **C** understanding the signs of life.

In 2020, the astrobiology and astrochemistry science community in Europe was tasked by ESA to provide an up-to-date scientific roadmap for the utilization of current and future space platforms (ESA SciSpacE Science Community White Papers: esamultimedia.esa.int/docs/HRE/SciSpacE_Roadmaps.pdf). This work was supported by ESA and builds upon work by previous ESA topical teams and experts in the field who extensively reviewed the scientific literature and the possibilities to advance our knowledge and understanding in astrobiology and astrochemistry research^3,14,15^. In addition to ground-based research, platforms, and concepts for experiments in space have been explored and discussed. To best utilize such space platforms, a number of top science objectives and related sub-objectives were identified. The interdisciplinary nature of this field prevents prioritization among these closely interwoven topics. Figure 1 shows the main themes and key areas that have been recognized and agreed upon. They consist of (A) Understanding the origins of life, (B) Understanding habitability and the limits of life, and (C) Understanding the signs of life. Each key topic and its sub-topics are described in more detail in the following sections:

### The origins of life—topic A

Life on Earth is currently our only accessible and scientific comprehensive reference for astrobiology studies. How life originated on Earth is a central question to inform and guide our search for life beyond our planet.

While the Earth’s environment, with stable liquid water at the surface, is unique in the Solar System today, this was not always the case. When life emerged on Earth (maybe more than 4 billion years ago), the environment was more similar to that of the other early rocky planets in the Solar System, such as Mars or perhaps Venus. Similarly, subsurface liquid water is present on icy moons such as Europa or Enceladus. As these environments resemble subglacial Antarctic lakes found on Earth (which are known to harbor a diverse assemblage of microorganisms^16^), icy moons are highly interesting candidates in the search for life. Despite these similarities, beyond our solar system and among the expanding number of known exoplanets, an analog of either the early Earth or the Earth today has yet to be found. It is crucial to understand the composition and role of the primitive atmosphere and the lithosphere (e.g., organic synthesis in hydrothermal vents), as well as the role of solar radiation, taking into account a different atmospheric composition than today’s and a faint young sun. These are all critical factors to be addressed in assessing both the specific and general roles of the Earth’s environment.

It is now commonly accepted that a significant part of the organic material in early Earth’s environment was provided via meteorites and micrometeorites, originating from carbonaceous asteroids and comets^17–19^. It is therefore important to understand the origin and formation of such material and how the journey through space influences organic material before it was delivered to Earth. With this in mind, various questions arise regarding the role of exogenous organic material delivery by small cosmic bodies, including but not limited to: where and how was the organic matter formed and how was it incorporated into small bodies or planetesimals; how radiation affects the formation of organic compounds; how much of this material was delivered to Earth; how might the mineral matrix of the small bodies change during space travel; how the physicochemical properties of inorganic and mineral surfaces may have affected the formation, nature, preservation, amount and local distribution of organic material; do these factors play a protective role against space radiation or atmospheric entry; and what is the significance of exogenously delivered organic material versus endogenous organics in the prebiotic chemistry leading to the origins of life^20^?

A key point in studying the origin of life is to understand abiogenesis; the transition from purely chemical, to a molecular prebiotic phase, and finally to a living and replicative system. Defining life as an auto-replicative system that evolves by natural selection, we can state that chemistry naturally spawns biology. Major improvements have been achieved in this field in the past few years^21^. Organic chemistry and chemical evolution are clearly central to this integrated understanding.

### Habitability and the limits of life—topic B

Study of life on Earth has shown the astounding ability of living systems to adapt to the most extreme and improbable environments on Earth (withstanding extremes in temperature, pressure, pH, humidity, salinity, radiation dose, and oxidation)^22,23^. In fact, the majority of terrestrial environments are inhabited by multiple domains of life. The emergence of life on Earth under environmental conditions very different to those reigning today, and the tremendous capacity of life to adapt to and even prosper under conditions that we would consider “extreme”, provide perspective for the search for life elsewhere in the Solar System, and broaden the scope of what the term “habitable” can mean.

Many inhabited extreme environments on Earth combine several parameters that are considered (from a human perspective) to be extreme in themselves. The resistance of a given organism to environmental extremes (either naturally occurring or artificial), and how these potential stress-inducing parameters influence its overall response, is an important avenue of research. Some studies of the effects of individual and combined extreme environments can be performed at appropriate field sites on Earth or using simulated environmental parameters in the laboratory^24^. However, to truly examine the combination of effects induced by the complex space environment, space experiments are a necessity. As such, ground and space-based approaches should be seen as complementary to maximize scientific return. Our improved understanding of characteristics, mechanisms of adaptation, and resistance of astrobiologically relevant and extremophilic organisms to space conditions is critical in understanding the biological effects of the space environment. Such studies are needed to define habitability (for life as we know it) and to support space exploration and the search for life beyond our planet. This includes continued protection of humans in space, as well as protecting the Earth, other planets and moons as human space exploration progresses.

Despite the broad range of physically and chemically extreme environments naturally present on Earth, some extraterrestrial conditions are specific to space, planetary, or planetary satellite environments. These conditions include low pressure down to space vacuum, exceptionally low relative humidity, micro- and fractional gravities, or parameters that may mimic the early Earth environment (anoxic, high radiation, warmer temperatures). Moreover, the Earth’s present magnetic field and atmosphere attenuate the far higher doses of ionizing and short-wavelength solar ultraviolet (UV) radiation that exist in space or on the surfaces of some other solar system bodies. It is possible to mimic single components of space radiation on Earth, but, due to its complexity, the radiation field in space can only partly be simulated^25,26^. Access to space environments is necessary in order to perform in situ exposure of organisms and their component macromolecules (nucleic acids, carbohydrates, lipids, proteins, etc.). This allows for the measurement of space-radiation-induced metabolic, genetic, and phenotypic changes, as well as the survival of, or damage to, key biomolecules. When investigating the constraints of life beyond Earth, such space-based experiments are critical in identifying individual or combined physically extreme parameters, that cannot be found or simulated on Earth.

Terrestrial organisms typically form groups and communities that provide advantages for survival and adaptation to environmental conditions. Single organisms may not be able to cope with extreme environmental parameters; however, biological interactions may provide collective protection, thus protecting many individuals. Adaptation to large environmental changes on a planetary scale (such as those that occurred on Mars^27^) may be mitigated on the micro-scale in environmental niches by associations of organisms of either the same or different species (dual/multi-species biofilms, symbionts, ecosystems^23,28,29^), and their interactions with abiotic material (rock/regolith layers, etc.). Investigating survival and adaptation strategies based on community formation and symbiotic relationships is important to understanding the limits of habitability.

As there is an increasing number of organisms being discovered and described that show adaptations to extreme environments^30,31^, it is essential to determine if these novel extremophiles imply that life could be distributed (either naturally or artificially) through the Solar System. There is a possibility that organisms could travel to and survive within interplanetary meteorites (e.g., those ejected from Mars to Earth); the mineral protection and preservation of organisms or biomolecules under these relevant environments is important. In addition to the natural distribution of life in the Solar System, we must assume that space exploration could result in forward contamination of solar system bodies by terrestrial material. This further underlines the need for Earth orbit and space-based in situ experiments, focusing on the survival strategies of organisms and their means of adaptation to environmental parameters not found on Earth. Knowledge of these survival strategies and the limits of extremophilic organisms will lead to further developments and improvements of decontamination procedures in a context of planetary protection. Currently, such decontamination procedures are the only way to minimize the risk of contamination of other worlds with terrestrial life. This is of particular importance for destinations that are considered habitable and may have (developed) their own biota. The investigation of viable spacecraft microbiota (both external and internal) will support more targeted, destination-dependent planetary protection measures. The potential impact and likelihood of forward biocontamination by both robotic and human missions must be considered very carefully, both at the technical and operational level, particularly assessing their compatibility with life-detection missions.

### The signs of life—topic C

To understand the signs of life (biosignatures) in and beyond our solar system, we must focus on cells, their remnants, clearly cell-related biochemical molecules, as well as biomediated structures. In addition, studying environmental transformations (including potential bio-driven transformations of atmospheric composition) should be an important objective. This topic focuses on detecting signs of life using a suite of complementary instruments, on the characterization of distinct cellular components and the stability of these biomolecules, as well as any specific physical evidence of interaction of cells with their environment. This is particularly relevant for in situ missions searching for evidence of life, as well as for analysis of returned extraterrestrial samples.

The search for signs of extinct life (either biomineralized or fossilized) relies on detecting organic, geochemical, isotopic or morphological remnants or other biomediated phenomena, such as biolaminae or stromatolitic bioconstructions^32^, at, or within planetary subsurfaces or ices. A better understanding of the process of fossilization and how extant biosignatures are preserved over geological time is required^33^. A relevant example of this is the search for past life on Mars. UV radiation interactions with organic remnants under different atmospheres, temperatures, pressures, and humidities needs to be investigated using references, such as terrestrial fossils from Earth. This can be performed under simulated conditions (planetary-simulation facilities^34^), however space-exposure facilities provide access to environmental parameters not available on Earth, such as microgravity, full-spectrum solar and cosmic radiation. Such experiments can provide insights into early-Earth conditions similar to those expected on rocky planets (such as Mars or Venus) or exoplanets.

Looking beyond our solar system, the simulation of potential exoplanetary conditions is crucial to decoding spectral signatures and therefore to understanding and interpreting their formation and evolution. Space-based experiments are of particular importance when assessing the impact of the solar spectrum and cosmic rays on biological compounds and organisms as fully representative photon and particle spectra cannot be reproduced in the laboratory. As molecules produced by life forms might not be unambiguously identified, this is a particular challenge for remote detection in (exo)planetary atmospheres or surfaces. Therefore, it is paramount to understand transformational processes and biochemical pathways of biosignatures, especially those that can be detected as volatile organic compounds or gaseous biosignatures in a planetary atmosphere. Also, with the various types, ages and sizes of stars, different planets receive different stellar spectra of electromagnetic radiation. Therefore, to identify signals of potential pigmented life forms on exoplanets, potential biochemical pathways of ultraviolet-visible (UV–vis) absorbing complex organic molecules synthesized in response to spectra different than that of the Sun should be investigated.

In the search for extant life, it is important to analyze biomolecules that can serve as cellular constituents. These include amino acids, peptides, lipids, pigments, and carbohydrates. Additional biomolecules of importance are those known from Earth organisms such as sterols, quinones, and porphyrins, as well as polymeric biomolecules that can store and transfer information, e.g., genetic material and proteins. With the assumption that non-Earth life is similar to life as we know it (i.e., based on carbon-bearing molecules with water as a solvent), it is possible to characterize cellular life cycles based on the presence, amount, or change over time of potential biomarker molecules on a planetary surface or atmosphere. In addition to environmental processes influencing biomarker molecules, the converse may also be true, with cellular processes having the potential to influence their surroundings. Such a reciprocal influence must be accounted for when classifying biosignatures or identifying extant life in a space or planetary environment.

A systematic approach for the detection of microbial life forms can be based in part upon the collection of data from the known microbial world. Environmental parameters and the geological evolution of potential host planets or planetary bodies should determine their viability as search targets. For instance, life-detection missions to Mars and to the moons Europa and Enceladus will include means to seek signatures of microorganisms similar to terrestrial life forms with metabolisms that could have been present on early Earth (including chemotrophs/anoxygenic photosynthesizers/certain heterotrophs^35^). Similar evidence-based considerations are needed to tailor missions to other bodies in our solar system. Furthermore, studies are needed to understand the relationship between the signs of life and various environmental conditions present at planetary-analog field sites, during planetary-simulation experiments, as well as in space. Each of these environmental parameters could alter or hide biosignatures, or produce false positives, such a minerals or organomineral structures that imitate the relatively simple morphology of microorganisms. These approaches should not only account for biosignatures derived from “life as we know it” but should also include agnostic biosignatures, i.e., signs of chemical or geological disequilibrium. As such, a variety of detection instruments and analytical techniques should be utilized to systematically add to existing databases such as NASA’s Astrobiology Habitable Environments Database. The integration and synchronization of centralized spaceflight experimental data repositories is a necessity in the future.

## Space platforms for astrobiology and astrochemistry

### Why space experiments?

Space provides a unique environment for performing astrobiology and astrochemistry experiments. Ground-based research is useful for studying the impact of environmental factors on the origin and evolution of life on Earth, and typically provides access to standardized reproducible conditions allowing quick repetitions of experiments, larger samples sizes, higher sample numbers, precise control of physicochemical parameters and an increase in the variety and resolution of analytical techniques at typically lower cost, when compared to space-based experiments. However, ground-based research can currently only be used for assessing single (or a limited sub-set of) space-based environmental factors, and as such provides only limited information on the combined influence of these factors. Experiments performed in space allow the study of effects induced by microgravity, by the wide spectrum of photons and energetically charged particles, as well as their combined effects on samples to be studied. To gather a complete and robust picture of influence of the space environment, a complementary approach must be utilized, exploiting the strengths of both in situ experimentation and ground-based research.

Within the context of searching for signs of life, the rationale for missions with the aim of visiting other celestial bodies (e.g., Mars) is mostly self-evident; however, remote-sensing platforms must also be tested and implemented. In addition, space-based experiments that focus on cellular life cycles, adaptation, biomineralization and fossilization processes must often be complemented by diverse ground-based experiments.

**Fig. 2:**
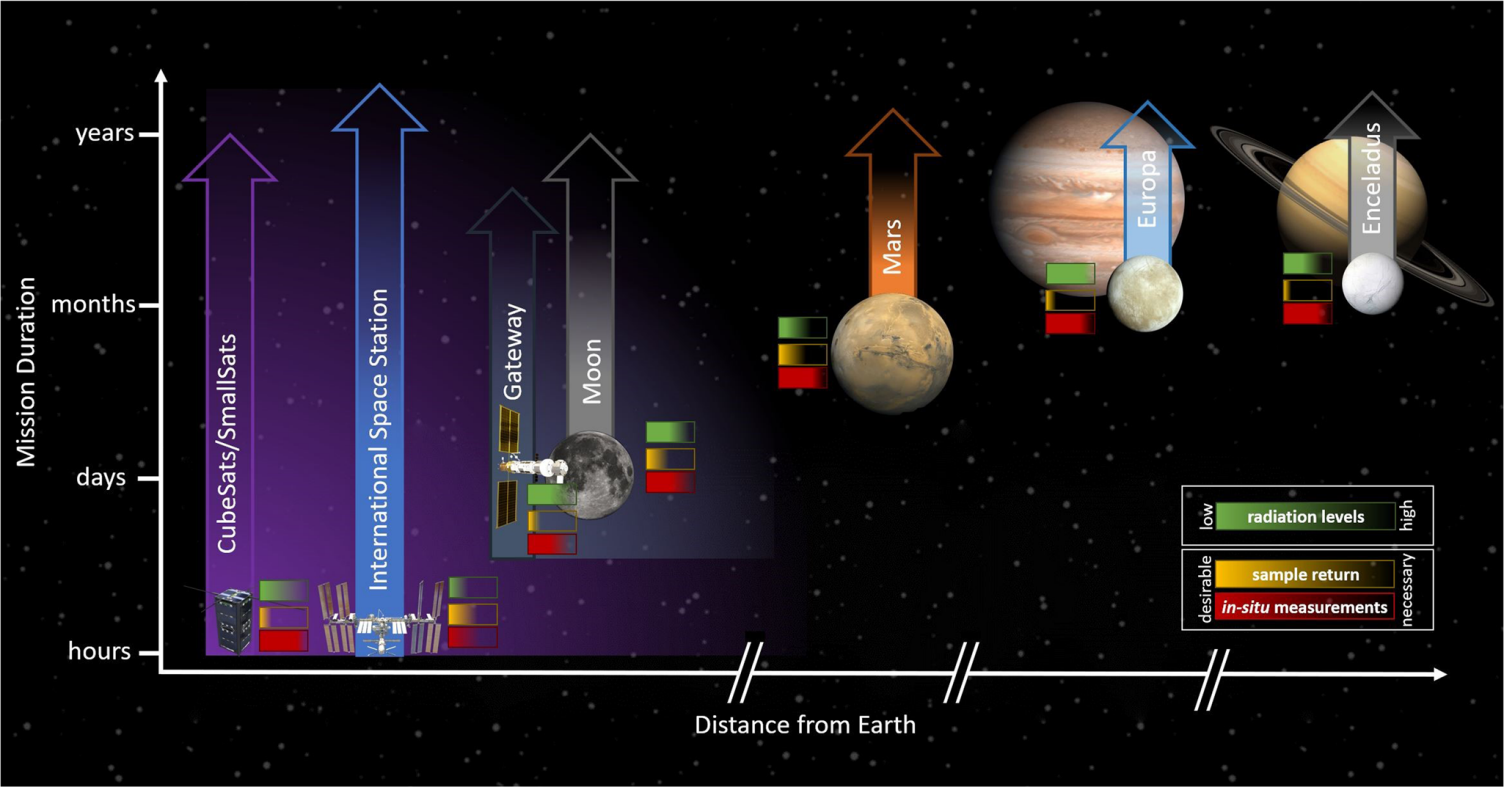
Space platforms for astrobiology and astrochemistry research. Space exposure experiments require suitable platforms for providing levels of radiation and microgravity. Platform location dictates mission duration, radiation exposure, the potential for sample return and the necessity of in situ measurements. As the distance from Earth increases, different radiation environments become available at the cost of increasingly challenging sample return.

Commonalities and properties of existing and planned platforms have to be identified to better define the experimental requirements and limitations of specific space platforms, and their suitability for astrobiology and astrochemistry experiments must be assessed. With this assessment, it is possible to decide how best to utilize space experiments to address key astrobiology and astrochemistry topics. Figure 2 illustrates potential locations for a number of space platforms, their distance from Earth and the potential range of mission durations. The distance from Earth and the mission duration give an initial indication of the possibilities of these platforms and are important characteristics for various astrobiology and astrochemistry experiments. For example, distance and duration are correlated with the type and amount of radiation that targets would receive.

### Why experiments in specific orbits/locations?

Space-based experiments in certain low Earth orbits (LEOs), or on the Moon and Mars, allow access to higher fluxes of high-energy photons, galactic cosmic rays and solar energetic particles compared to the terrestrial environment. Specific locations, however, can have vastly different radiation levels. For example, the Moon receives a very high radiation dose whereas the level on Mars is lower due to its thin atmosphere. To constrain radiation-driven processes and examine their effects on biology, simultaneous ground-based and space experiments are needed. An advantage of space experiments, especially in the field of astrochemistry, is that platforms can be designed and operated far from sources of terrestrial or artificial contamination (e.g., atmospheric pollution, outgassing events from larger platforms, vibrations and electromagnetic interferences). Similarly, remote-sensing methods that use telescope optics to collect spectral data in the near-infrared (IR) to radio wavelength ranges, as well as visible and UV, function most optimally beyond the Earth’s atmosphere.

Within the context of finding signs of life in our own solar system, it is clear that sending probes to planets and moons of interest is the most efficient way to search for signatures of extant or extinct life. The nature of these missions is highly dependent on the environment of the world under investigation. For example, in the case of Europa, current projects are solely orbital, relying on remote-sensing techniques as well as encountering ejecta from the surface of the moon itself^36,37^, even though mission proposals for in situ investigations are discussed. On the other hand, previous, current and future missions to Mars and Titan include significant landing modules to study the surface directly^38,39^. A special case, Saturn’s moon Enceladus ejects ice particles from its subsurface ocean into space via south polar “cryovolcanoes”, providing fly-by missions the opportunity to examine recently frozen water for life’s signatures^40^.

### How long would the mission duration need to be?

In order for large-scale space-based facilities (e.g., the ISS, the James Webb Space Telescope, or the planned PLATO spacecraft^41^) to make fiscal sense, their lifetimes must be typically on the order of decades. However, the advent of SmallSats and CubeSats (e.g., O/OREOS^42^, SpectroCube^7^, IR-COASTER^5^, BioSentinel^8,9^) are currently challenging this assumption. Short-term exposure experiments (e.g., BIOPAN^43^) should be used as predecessors or viability assessments for long-term exposure experiments, and small-scale missions (e.g., Twinkle^44^, CUTE^45^) should be used to support multi-decade lifespan spacecraft. This implies that miniaturization of existing technology is of the utmost importance.

The study of photochemical processes and reaction pathways typically requires several months of radiation exposure in, for example, LEO to accrue a total radiation dose that produce measurable effects, leading to overall mission times on the order of one year. Similarly, radiation-biological effects on some extremophilic microorganisms require months of exposure to accumulate. However, this must be assessed based on the tolerances of the organism under investigation, as well as the specific location of the experimental platform. Finally, to assess the long-term, cumulative effects and adaptations to space radiation, radiotolerant and extremophilic organisms (and organisms with resistant forms, e.g., spores) should be exposed for long durations. The importance of time taken to reach a destination becomes even more important for missions further afield, such as to Mars or the moons of Jupiter and Saturn. In these cases, there is a minimum mission duration ranging from months to a decade or more with current propulsion technologies.

### What mode of operation is required?

In the past, space-exposure platforms have relied on sample-return experiments (e.g., the Long-Duration Exposure Facility^46^, EURECA, EXPOSE-E, EXPOSE-R, EXPOSE-R2^47–50^), and while such methods provided access to the LEO environment, the lack of time-resolved data was a major limit to the conclusions that could be drawn. The collection of data during space missions is highly desirable for future space experiments. Such in situ analyses are an important way of adding redundancy and reducing the risks of space missions, while at the same time providing a more detailed, comprehensive data set compared to experiments relying solely on pre/post-flight analysis.

When designing future space facilities, organic compounds of prebiotic interest, cellular and molecular biosignatures, as well as both the fossilized remains and live microorganisms should be studied under plausible space and planetary conditions, with variable but known and controlled radiation, pressure, and temperature parameters^51^. To investigate a wide range of environmental parameters, space-based experimental facilities should implement dynamic humidity levels, wet/dry cycling and freeze/thaw cycles with the possibility of real-time analyses to follow any changes encountered. New facilities should allow for in situ thermal control and the possibility to simulate cool planets (e.g., N_2_ cooling cycles), icy moons, comets, and interstellar-medium conditions (e.g., He cooling).

For experiments involving living organisms, multiple generations of live, metabolically active organisms should be exposed via small payloads that implement fine temperature control, relative humidity, pressure, pH, atmospheric composition, nutrient/reagent supply, and the removal of waste products (liquids and gases). Bioreactors and microevolution chambers require further development and optimization, for instance microfluidic systems can implement fine control of a variety of environmental parameters. Microwells, each with independent fluidic inlets and outlets, can be utilized for a large number of low-volume microbial growth experiments, operated in parallel^52^. Experiments with living systems (such as NASA’s BRIC^53^, BioCell Habitat^54^) require automatic assay, often including subsampling at regular intervals, in situ telemonitoring (observing the appropriate functions, e.g., metabolism, genetic transcription and translation, self-repair mechanisms and quantification of adaptions), and the capacity for adjustments to be made via telecommand.

Although in situ analysis is currently the most effective means to acquire data from interplanetary probes, sample return or (ex situ) lab analogs are highly informative and complementary to such in situ analyses. To learn the most from in situ biological experiments conducted in space or planetary environments, following exposure, it is highly desirable to preserve and return samples to Earth for in-depth, laboratory-based studies. Of particular interest is the genomic, proteomic, transcriptomic & metabolomic influence of the space environment. This will require standardization of experimental protocols for selected, well-studied model organisms of interest, allowing gathered in space to be compared between experiments data.

In general, analytical techniques should have a dual function: on the one hand, to give extensive information on the processes at work, and on the other hand, to allow comparison with astronomical data and data from space missions^38,55^. In addition to experiments focusing on the exposure of samples to the space environment, methods must be designed to process and handle samples returned from space missions, with particular emphasis on planetary protection and life detection. In this regard, space platforms with frequent access (e.g., ISS) are ideal to test sample-return scenarios for interplanetary missions (e.g., Mars).

### Which (in situ) analyses are foreseen?

While several biological methods and technologies have recently been adapted to space conditions with operation by human crew (e.g., DNA extractions^56^, the FLUMIAS live cell imaging microscope^57^, and RT-PCR instruments^58^), to fully understand the scope and details of the impacts of extended durations in the space environment upon terrestrial organisms, it is imperative to continue advancing space-compatible cellular analytical techniques, such as qPCR^58^, high-throughput sequencing, fluorescence-activated cell sorting^59^ or sub-cellular microscopic techniques. In addition to studies of monocultures of extremophilic microbes, biological interactions, such as biofilms, symbionts or microbial communities may result in increased resistance to the environmental stressors of space. As such, focus should be placed on understanding how a given biological interaction influences survivability and adaptation, along with the identification of keystone species that are particularly influential. For a detailed analysis of such community samples, both in situ and postexposure analyses (with instrumentation not available for in-flight measurements) are typically required.

In order for landed missions, such as the Mars surface rovers (mars.nasa.gov/msl/, mars.nasa.gov/mars2020/) to aqcuire evidence that could point to (extant or extinct) extraterrestrial life and to more generally understand the organic chemical history of other bodies in our solar system, they require sophisiticated in situ measurement and data analysis capabilities. Samples such as rock cores can be examined in both a geological context and in the search for organic molecules, having the potential to provide information on the decay of biomarker molecules or life cycle processes. To obtain such information, landers and rovers use a range of observational techniques (surface imaging via radar, cameras and microscopes) in combination with various in situ spectroscopic and spectrometric methods. Gas chromatography mass spectrometry (GC-MS) is a cornerstone in situ analytical technique for landers^60^ and is currently the only way to detect enantiomeric excess of chiral molecules in situ^61^. Prominent examples of its use on Mars include the Viking missions of the mid 1970s^62^ and the Mars Science Lander^63^ that continues to operate on the Martian surface. Sample mapping and composition analysis is commonly performed using a variety of spectroscopic techniques, including UV–vis absorption and UV–vis fluorescence measurements, transmission and reflection Fourier-transform IR microscopy, Raman, Mössbauer, X-ray diffraction and X-ray fluorescence, and laser-induced breakdown spectroscopy^7,42,64–66^. Other useful techniques for analyzing both organic and inorganic species, currently being miniaturized for in situ use include laser ablation and laser ablation ionization mass spectrometry^67,68^.

The current technologies outlined above are being utilized for specific selection of candidate samples, which can later be returned to Earth for more detailed study (e.g., the current Mars Sample Return campaign^69^). However, looking to the future, more advanced in situ techniques could be miniaturized and implemented, potentially alleviating the need for sample return and thus reducing overall mission complexity and cost. For example, in situ MS is a very relevant analytical technique, recently used by the Mars Science Laboratory^70^ to analyze gases and sublimated species released by thermal means. However, heating a sample to high temperatures may release volatile organic species that can trigger chemical reactions or the degradation of potential biomarkers. As such, new technologies are being developed to extract soluble organics from solid (irradiated) samples at mild temperatures using solvent-based techniques, without degradation^71^; following such extraction, various high-resolution MS and tandem-MS techniques can be employed to understand the nature and provenance of the organic signatures by measuring structural information as well as the extent of the “decay” (alteration over time) of molecular structures.

### Which platform would be best suited?

The launch and maintenance of large-scale space platforms (such as space-based telescopes or manned platforms) require huge, dedicated, often multinational, space agency missions. The ISS remains an important exposure platform for both short- and long-term experiments, with the possibility for sample return. Furthermore, the ISS can be utilized as a test platform for future developments and the technological heritage from the ISS can be re-utilized on other platforms. The Lunar Gateway is progressing toward hosting such experiments in the Moon’s vicinity in a matter of years; nonetheless, nanosatellites, CubeSats, and SmallSats are becoming increasingly robust and readily available. They have proved capable of providing complementary information and thus are opening the field of study in this regard (e.g., Pandora^72^). SmallSats allow for studies ranging from the time-dependent alteration of molecules exposed to particle and electromagnetic radiation, to mimicking conditions on small bodies, to studies of the impact of the space environment on organics in meteorites, and they are showing that astrochemistry exposure experiments can be done outside of traditional platforms such as the ISS. These platforms are potentially also well suited for space biology experiments that expose living organisms over multiple generations to microgravity in combination with levels and distributions of energetic particle radiation only available beyond LEO. To execute such studies effectively the experimental durations aboard these small platforms need to be extended to (many) months to accumulate total radiation dosage with measurable biological effects. As mentioned previously, the continued development of highly sensitive and sophisticated autonomous bioanalytical systems with potential to measure genetic parameters, -omics, and other key biological properties is required.

Nevertheless, life-detection experiments requiring surface landers continue to require costly, dedicated missions. For a lander to make an unambiguous set of measurements that either support or refute a finding of the presence of life on another world, multiple complementary and synergistic analytical methods will most likely be required. As such, larger-scale platforms (relative to CubeSats and SmallSats) are required in this scenario, first to allow for landing, and second to house the required suite of sophisticated analytical tools that are typically too bulky for small platforms. A drawback of SmallSats is the lack of sample-return capabilities in most cases, which is necessary to investigate a variety of cellular effects on ground with a suite of sophisticated instruments not (yet) available in and beyond LEO.

Living metabolically active organisms, cellular processes or community composition may be directly influenced by non-Earth gravity (either micro- or hypergravity). Direct, in situ investigations (using exposure platforms) will solve many issues associated with simulated gravity experiments^73^. Similar experiments with high fluxes of galactic cosmic rays and solar particles are required, especially in preparation for human exploration. A main focus of space platforms is the combined influence of microgravity and varying space-radiation conditions. These can be compared against laboratory facilities (clinostat, simulated solar radiation, gamma radiation sources, heavy ion accelerators, electron beam facilities, X-ray sources, etc). Additionally, new space facilities (such as the Lunar Orbital Gateway) will provide a novel environment in which the establishment of a new microbiome can be studied. While a similar capability for a more limited class of experiments is in principle also feasible with SmallSats or CubeSats, the Lunar Orbital Gateway will be distinct, given the limited human presence and potential for long-term monitoring. A “clean” and isolated environment such as this is unique, and thus monitoring of the microbiome over time could provide valuable insights and important information for future habitats on the Moon or Mars.

In addition to focusing on changes induced by the space environment, experiments to determine the transformative effects from the process of re-entry have also been performed^6,74,75^. Samples such as Martian sedimentary rocks containing organic material have been placed in the heat shield of craft returning from LEO, subjecting them to extreme temperatures and a high-velocity plasma environment that is incredibly challenging to replicate ex situ. The survivability of organisms and/or the degradation of biomolecules should be assessed under the extreme pressure and temperature conditions of atmospheric re-entry and surface impact. Such experiments should be conducted on platforms (e.g., STONE^76,77^), re-entry nanosatellites, or as external additions on larger returning spacecraft. The potentially protective influence of rocky body-associated minerals must also be accounted for. With regard to both forward (contamination from Earth carried to other bodies) and reverse (extraterrestrial organisms brought back to Earth) planetary protection, both internal and external biocontamination must be assessed at the molecular level; a process that is also mandatory for search-for-life missions in order to eliminate serious risk of false positives.

### Recommended space-exposure payloads: short and medium term

The key questions in each of the topics presented in the introduction of this perspective can be addressed by specific space experiments on either multi-experiment space-exposure facilities or by means of tailored space platforms. Short (next 3 years) and medium (next 5 years) term recommendations for experiments in the key area A, “understanding the origins of life” include the design and implementation of experiments with active analytical capabilities (e.g., in situ spectroscopy and mass spectrometry) and active environmental control. In particular, platforms capable of maintaining sample temperatures well below 0 °C, ideally even at temperatures as low as <100 K, are required for a next generation of astrochemistry experiments and investigations of ice-organics mixtures, icy-moon, and interstellar-medium conditions. A further recommendation is to perform such experiments in locations with minimal terrestrial pollution, for example avoiding outgassing events from larger facilities such as the ISS. In addition to exposure experiments, platforms designed for re-entry into the Earth’s atmosphere are recommended to advance our understanding of meteoritic impact processes. Such platforms should be capable of carrying and analyzing samples either in situ or after hardware retrieval.

In key area B, “understanding habitability and the limits of life”, recommended experiments and platforms should focus on the impact of the space environment on living (micro)organisms, in particular, the combination of radiation and microgravity. Paired with in situ analytical capabilities, these experiments should study the response and adaptation of living systems to multiple stresses that can be monitored directly in space. This will likely require microfluidic and liquid-handling systems that function reliably in space. In addition to in situ data, sample return for in-detail analysis after the space-exposure phase is highly desirable. Re-entry platforms are recommended for the study of impact scenarios and how they affect either actively-growing or dormant living organisms.

With the focus of key area C, “understanding the signs of life”, being on detectability and identification of potential biosignatures, experiments in this area must be capable of simulating space and planetary conditions, including the respective radiation environment (electromagnetic and particle radiation). This can be achieved by designing and implementing space-exposure platforms that can access specific radiation environments, e.g., low- or highly elliptical orbits around Earth, the moon or interplanetary platforms. In situ analysis will be a key tool for such experiments investigating the stability or alteration of specific biosignatures (in the solid or gas phase) under conditions mimicking space and (exo)planetary conditions.

## Future outlook and summary

We live in exciting times for space sciences and space exploration with an unprecedented number of missions in the implementation or planning phases. Driven by commercialization and reduced launch and development costs, the progress in space technology, miniaturization and automation offers new possibilities for experiments in space environments and enables the design and implementation of new space-exposure platforms. With the advent of artificial intelligence, machine learning and robotics, performing more complex scientific experiments beyond Earth is becoming increasingly feasible and enables addressing important questions in the fields of astrobiology and astrochemistry, highlighted in the introduction to this perspective. Astrobiology experiments with live cells require sophisticated fluidic systems and in situ analytics and are key to advancing our understanding of the limits of life on and beyond Earth (topic A). The space environment, for the exposure of samples to early Earth or other simulated planetary conditions, is an excellent tool for addressing questions in relation to the origin and evolution of life, including prebiotic chemistry (topic B). Astrochemistry aims to investigate processes and conditions not necessarily found on Earth and difficult to simulate in terrestrial laboratories. While ground experiments can provide a cost-effective means to acquire preliminary data in preparation for space experiments, having access to space environments via the space platforms discussed is critical to perform astrochemistry experiments in their native environments thus allowing the simulation of important astrophysical, astrochemical and planetary conditions more faithfully than Earth-based facilities. This will be crucial for understanding the formation of organic molecules as well as potential biosignatures, and in support of current and upcoming life-detection missions to planetary and lunar bodies in our solar system (topic C). Beyond the solar system, and in the rapidly expanding field of exoplanetary sciences, new space platforms and telescopes play a pivotal role in understanding planetary habitability and possibly detecting signs of life, which has the potential to revolutionize our understanding of life in the universe.

### Reporting summary

Further information on research design is available in the Nature Research Reporting Summary linked to this article.

## Supplementary information

Reporting Summary
